# Selective control of primer usage in multiplex one-step reverse transcription PCR

**DOI:** 10.1186/1471-2199-10-113

**Published:** 2009-12-30

**Authors:** Elena Hidalgo Ashrafi, Joyclyn Yee, Natasha Paul

**Affiliations:** 1 Department of Research and Development, TriLink BioTechnologies, Inc., 9955 Mesa Rim Road, San Diego, CA 92121, USA

## Abstract

**Background:**

Multiplex RT-PCR is a valuable technique used for pathogen identification, disease detection and relative quantification of gene expression. The simplification of this protocol into a one-step procedure saves time and reagents. However, intensive PCR optimization is often required to overcome competing undesired PCR primer extension during the RT step.

**Results:**

Herein, we report multiplex one-step RT-PCR experiments in which the PCR primers contain thermolabile phosphotriester modification groups. The presence of these groups minimizes PCR primer extension during the RT step and allows for control of PCR primer extension until the more stringent, elevated temperatures of PCR are reached. Results reveal that the use of primers whose extension can be controlled in a temperature-mediated way provides improved one-step RT-PCR specificity in both singleplex and multiplex reaction formats.

**Conclusions:**

The need for an accurate and sensitive technique to quantify mRNA expression levels makes the described modified primer technology a promising tool for use in multiplex one-step RT-PCR. A more accurate representation of the abundances in initial template sample is feasible with modified primers, as artifacts of biased PCR are reduced because of greater improvements in reaction specificity.

## Background

Reverse transcription PCR (RT-PCR), a protocol widely used to produce DNA copies of an RNA strand of interest, involves a lower temperature reverse transcription step followed by an elevated temperature PCR step [1]. RT-PCR reactions are typically separated into two separate steps. However, as the number of manipulations in a reaction protocol increases, so does the probability of contamination. Reduction of the two separate manipulation procedures into a one-step RT-PCR protocol provides a more streamlined, high-throughput technique that lowers the probability of contamination by minimizing the number of handling steps [2]. However, one-step RT-PCR protocols are not without inherent limitations and have often been found to be less sensitive than the corresponding two-step protocols [3-5]. One likely cause for this lower sensitivity is competing extension of PCR primers by reverse transcriptase [6] or DNA polymerase [7]. At lower, less discriminatory temperatures of reverse transcription, PCR primer extension may result in off-target amplification products such as primer dimer and mispriming products. To improve the specificity of RT-PCR, it is necessary to block such primer extension at lower temperatures.

RT-PCR is commonly used in routine molecular diagnostics as a tool for detection of genetic diseases [8]. Multiplex RT-PCR is an advanced application of this technique that allows for detection of two or more RNA targets in a single tube reaction. This approach has been used to rapidly detect viral subtypes [9-12] or to simultaneously identify different disease mutations [13]. Multiplex one-step RT-PCR protocols enable the detection of multiple RNA targets in a single reaction and allow for quantification of relative mRNA expression levels using real-time detection [1]. This technique requires extensive optimization because the use of multiple primer pairs in a single reaction increases the likelihood of primer dimer and mispriming product formation, which may interfere with the amplification of the desired products. Another pitfall of multiplex RT-PCR is the preferential elongation of certain targets, both at the cDNA synthesis and PCR amplification stages of the reaction, especially when the targets differ greatly in relative abundance [14]. These difficulties make multiplex one-step RT-PCR a challenging technique with rewarding results when successful.

To overcome the main drawbacks of one-step RT-PCR protocols, researchers have tried several approaches. One strategy is to employ Hot Start DNA polymerases. As Hot Start DNA polymerases are designed to only be active at the elevated, more stringent temperatures of PCR thermocycling, DNA polymerase-mediated formation of off-target amplicons during the less stringent temperatures of reverse transcription can be significantly reduced. Another approach to prevent primer-mediated formation of off-target amplicon during the cDNA synthesis step is the use of modified reverse transcriptases that are stable and active at higher temperatures, such as the SuperScript^® ^III RT (Invitrogen). By performing cDNA synthesis at higher temperatures, the binding of PCR primers to regions of lesser complementarity during the reverse transcription (RT) step should be reduced. However, since both the RT primer and PCR primers are present in the same reaction, there is the possibility of further complications due to extension of all three primers during the RT step. A different approach is to enhance one-step RT-PCR performance through the use of a physical barrier such as AmpliWax^® ^PCR Gems (Applied Biosystems). AmpliWax^® ^PCR Gems separate a single tube into two compartments, allowing for the RT step to occur independent of the DNA polymerase and PCR primers [15,16]. At the elevated temperatures of PCR, the wax melts to allow introduction of the DNA polymerase and primers into the RT reaction product. While this approach provides a means of further improving reaction performance in one-step RT-PCR, the preparation of the wax barrier presents an additional step that needs to be introduced into typical workflows. An alternative approach uses microfluidics to allow for the sequential introduction of primers in a reaction [17,18]. While this approach allows streamlined control over the introduction of the primer into the reaction this approach requires investment in highly sophisticated equipment. With these concepts in mind, it was of interest to learn whether blocking PCR primer extension during the RT step would afford a similar benefit.

**Figure 1:**
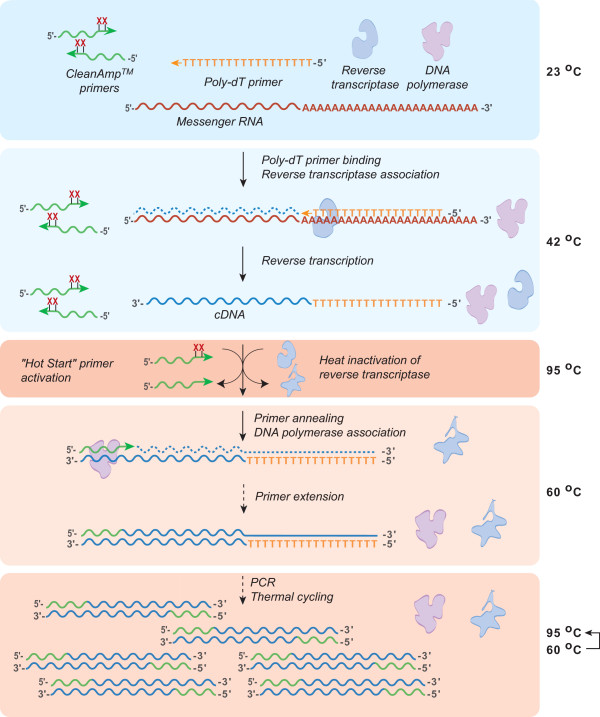
**Illustration of the benefit of thermolabile CleanAmp™ primers for selective primer utilization in one-step RT-PCR**. During the room temperature set-up and lower temperature reverse transcription steps, the presence of two thermolabile protecting groups (each indicated as X) blocks competing extension of the PCR primers. At reverse transcription temperatures, cDNA synthesis is performed using an unmodified oligo(dT)_18 _primer. A Hot Start activation step at elevated temperature (95°C) promotes loss of the thermolabile protecting group, which allows for use of the PCR primers during PCR portion of the thermal cycling protocol.

Herein, we will investigate the use of primers containing thermolabile protecting groups for improved multiplex one-step RT-PCR performance. Previous studies revealed that the introduction of two thermolabile phosphotriester protecting groups (marked as X in Figure 1) into the 3'-terminal and 3'-penultimate internucleotide linkages of a gene specific primer pair provided enhanced one-step RT-PCR performance [19]. The use of temperature-sensitive primer modifications aims to significantly reduce PCR primer extendability until the elevated temperatures of PCR are reached. This ability to block the extension of the PCR primers allows for the unmodified RT primer of choice, such as the oligo(dT)_18 _primer depicted in Figure 1, to be utilized during the cDNA synthesis step. The temperature-mediated removal of the thermolabile primer modification group allows for selective extension of the PCR primer pair during PCR thermal cycling. This study will build upon the early work of Lebedev et al. [19] to examine the use of two or more modified primer pairs in multiplex one-step RT-PCR protocols. The significance of this approach to one-step RT-PCR is great as researchers are more frequently relying on RT-PCR as the "gold standard" for accurate validation of microarray-based gene expression profile [17,18].

## Results

In these studies, the potential benefit of thermolabile PCR primers in multiplex one-step RT-PCR was investigated. In particular, one-step RT-PCR amplifications in this paper employed an unmodified oligo(dT)_18 _or random decamer primer for the RT step and one or more thermolabile primer pairs for the PCR step. An initial singleplex one-step RT-PCR experiment on three literature-defined targets [20] compared the performance of an unmodified PCR primer pair to PCR primers modified at both the 3' terminal and penultimate positions with thermolabile phosphotriester groups (CleanAmp™ Precision) [19] (see Figure S1 in Additional file 1). Results revealed further improvement using Precision modified PCR primers, regardless of the RT primer utilized. While the use of Precision primer modifications provided improved one-step RT-PCR specificity when an unmodified reverse gene specific primer was used, the greatest improvement in specificity was obtained when unmodified oligo(dT)_18 _or random decamer primers were used for the RT step. Based on these findings, a combination of Precision PCR primer modifications and an unmodified oligo(dT)_18 _or random decamer RT primer was selected for extensive evaluation in the following multiplex one-step RT-PCR experiments.

**Figure 2:**
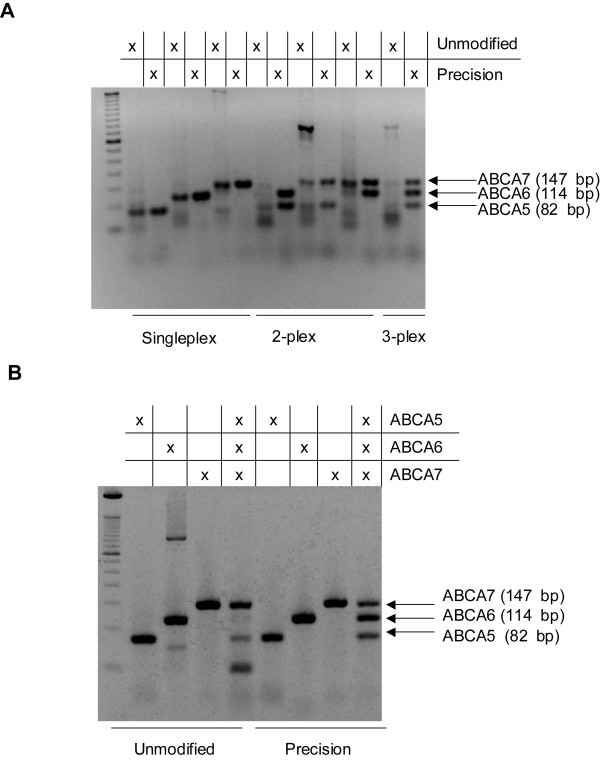
**One-step RT-PCR evaluation of unmodified and thermolabile CleanAmp™ Precision primers to amplify three different targets of ABCA transporters in singleplex, duplex, and triplex amplifications**. For each gene of interest (ABCA5, ABCA6, and ABCA7), the PCR primers were unmodified or contained CleanAmp™ Precision modifications. Reverse transcription utilized an oligo(dT)_18 _primer. Reactions contained *Taq *DNA polymerase, the appropriate reverse transcriptase, and 0.82 μg of human trachea total RNA. **A) **Reactions employed M-MLV reverse transcriptase and utilized an RT extension temperature of 42°C. **B) **Reactions employed SuperScript^® ^III reverse transcriptase (SSIII RT) and utilized an RT extension temperature of 55°C.

Three ABC transporter primer pairs for the human (Homo sapiens) ABCA5, ABCA6 and ABCA7 genes [21] were prepared as unmodified and Precision modified primers for examination of their performance in amplifying a single target. The previous studies of Langmann et al. [21] were expanded to multiplex reactions that simultaneously amplified two or three targets (Figure 2A). In a singleplex set-up, RT-PCR with both unmodified and Precision primers amplified the correct amplicon. However, reactions with unmodified primers yielded less amplicon, and the longest target (ABCA7), formed a higher molecular weight off-target amplicon. When all duplex combinations of the three targets were amplified, reactions containing unmodified primers were plagued by off-target amplification. In contrast, reactions containing Precision primers amplified all duplex combinations correctly with a lower level of primer dimer formation than reactions containing unmodified primers. When all three targets were amplified simultaneously, the use of Precision PCR primers showed high specificity for the targeted amplicons compared to the unmodified PCR primers.

Since it has been described that reverse transcription at elevated temperatures improves the quality of cDNA synthesis products and resultant PCR product quality [22], we next investigated whether an increase in the temperature of the reverse transcription reaction from 42°C to 55°C would improve the performance of reactions employing unmodified primers. A protocol using SuperScript^® ^III RT (SSIII RT), a reverse transcriptase that is stable at higher temperatures, was developed to amplify three targets individually and together as a triplex (Figure 2B). The amplification of single targets using SSIII RT with unmodified primers provided more specific amplicon formation than when the RT step was at 42°C, using M-MLV RT. However, simultaneous amplification of all three targets was poor, yielding robust formation of only the ABCA7 target, with lower yields of ABCA6 and ABCA5, and strong primer dimer formation. When the SSIII RT experiments were performed with Precision primers (Figure 2B), amplicon formation in both singleplex and triplex reaction formats was successful, providing similar findings as with M-MLV RT.

**Figure 3:**
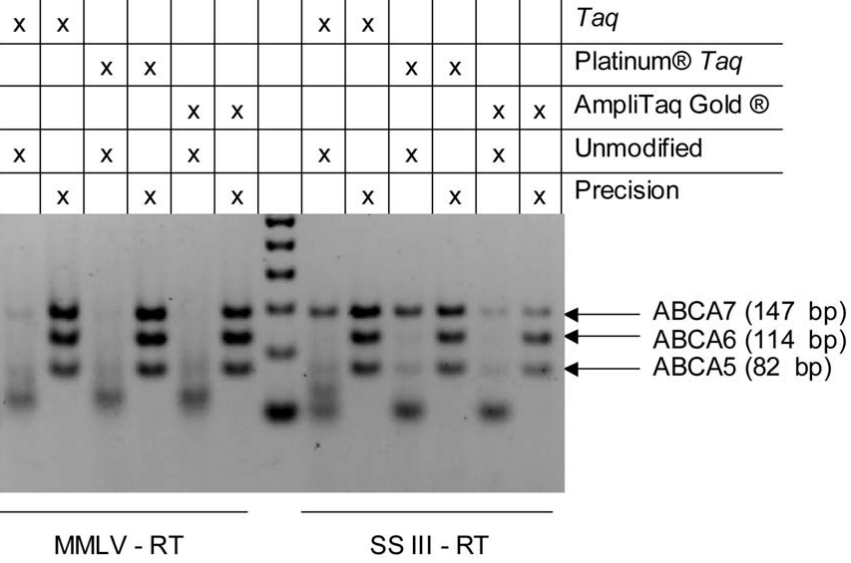
**Hot Start DNA polymerase evaluation in triplex one-step RT-PCR amplification of ABCA5, ABCA6 and ABCA7 targets**. Reactions contained 0.82 μg of human trachea total RNA and an unmodified oligo(dT)_18 _RT primer. The PCR primers were either unmodified, or contained CleanAmp™ Precision modifications. These reactions contained one of the following DNA polymerases: *Taq*, Platinum^® ^*Taq*, or AmpliTaq Gold^® ^and one of the following reverse transcriptases: M-MLV or SSIII. Reactions with M-MLV were incubated at 42°C, while reactions with SSIII were incubated at 55°C.

Next, *Taq *DNA polymerase was substituted with one of two Hot Start DNA polymerases [23,24] to determine whether formation of non-specific extension products was due to DNA polymerase activity at the lower temperatures of reverse transcription. In conjunction, we also wanted to investigate whether the use of a more specific reverse transcriptase, such as SSIII RT, would improve the specificity of the reverse transcription and subsequent PCR steps. Towards that goal, we evaluated triplex amplifications using M-MLV RT (42°C) or SSIII RT (55°C) as the reverse transcriptase, and *Taq*, AmpliTaq Gold^® ^or Platinum^® ^*Taq *as the DNA polymerase (Figure 3). When Hot Start DNA polymerases (AmpliTaq Gold^® ^and Platinum^® ^*Taq*) and unmodified PCR primers were employed, a slight improvement was observed with SSIII RT. However, amplicon yield was compromised by predominant primer dimer formation. The three DNA polymerases were subsequently evaluated with Precision primers to assess whether product enrichment could be further improved by such combination. For all combinations, the three targets were amplified with high specificity when modified primers were utilized, providing much better performance than with unmodified primers. There were indications of reduced amplicon yield with the slower-activating AmpliTaq Gold^® ^DNA polymerase [24], but still the yield was better than that obtained with unmodified primers.

The ability of Precision primers to support multiplex real-time one-step RT-PCR was next evaluated. To correlate copy number with real-time detection of RT-PCR products, RNA standards were prepared using by *in vitro *transcription of PCR amplified fragments of the ABCA5, ABCA6, and ABCA7 genes using the RiboMAX™ Large Scale RNA Production System-T7. These RNA standards were employed in subsequent experiments to allow for quantification of the number of copies of a gene of interest in total RNA or other RNA samples. For each real-time one-step RT-PCR experiment where unknown sample quantification was performed, triplicate serial dilutions of the ABCA5, ABCA6 and ABCA7 RNA standards were performed in singleplex. Dilutions of the RNA standards, ranging from ~10^1 ^to ~10^8 ^copies were robustly amplified, and produced resultant standard curves with efficiencies of 80-85% (Figure S2 in Additional file 1).

**Figure 4:**
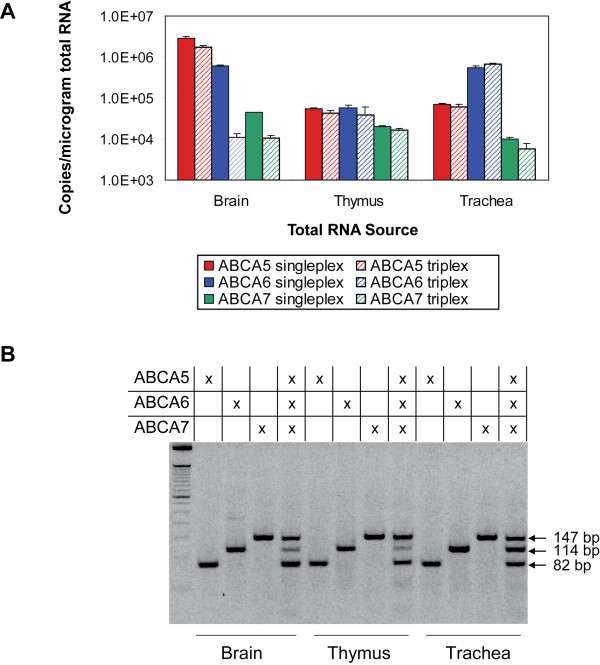
**Singleplex and triplex real-time one-step RT-PCR detection of ABCA5, ABCA6, and ABCA7 in three different tissues**. Reactions, which were performed in triplicate, contained M-MLV reverse transcriptase, an unmodified oligo(dT)_18 _primer, *Taq *DNA polymerase and CleanAmp™ Precision PCR primers for the ABCA5, ABCA6 and ABCA7 genes. A standard curve for ABCA5, ABCA6, and ABCA7 was determined by employing ~10^1 ^to ~10^8 ^copies of the appropriate RNA standard. Each of the three human total RNA tissue samples (brain (0.78 μg), thymus (0.8 μg), and trachea (0.82 μg)) was amplified in singleplex and triplex format for detection of ABCA5, ABCA6, and ABCA7. The number of copies of each target in a given tissue was determined by extrapolating the resultant Cq values to the standard curve and normalizing the resultant values to the micrograms of input total RNA. **A) **The relative number of copies per microgram and standard deviation for each target in brain, thymus, and trachea total RNA is represented in a bar graph, which displays the results for singleplex and triplex amplifications. **B) **The corresponding agarose gel analysis of the three tissue samples amplified in singleplex and in triplex.

Next, three human total RNA tissues were selected and amplified in singleplex and multiplex one-step RT-PCR experiments for detection of the ABCA5, ABCA6, and ABCA7 genes (Figure 4A). Each reaction, which amplified ~0.8 μg of total RNA, was performed in triplicate and was compared to the appropriate dilutions of each RNA standard. Results revealed high ABCA5 expression in the brain, high ABCA6 expression in the trachea, and very similar expression levels for ABCA5, ABCA6, and ABCA7 in the thymus. For almost all genes, the experimentally determined copy number was similar for singleplex and multiplex one-step RT-PCR experiments, with the exception of the ABCA6 gene in brain. This was likely due to a poorly-formed shape of the amplification curve for the triplicate reaction, which resulted in a large standard deviation in copy number determination for the ABCA6 gene (data not shown). Analysis of the amplification products by agarose gel electrophoresis (Figure 4B) revealed that the relative abundances calculated in real-time could be largely verified by endpoint analysis of the reaction products. To determine whether the amount of input total RNA would influence the resultant quantification of the sample, we repeated these studies using ~0.8, ~80, and ~800 ng of total RNA from trachea, thymus, and brain (data not shown). While the quantification cycle (Cq) values [25] determined in singleplex and multiplex assays agreed well with one another for a given input concentration of total RNA, we found that the amplification of the ABCA6 target was problematic, as it amplified with different efficiencies than ABCA5 and ABCA7. This finding is not uncommon in multiplex experiments and may be due to a number of factors, including preferential amplification when targets are significantly unbalanced [14].

**Figure 5:**
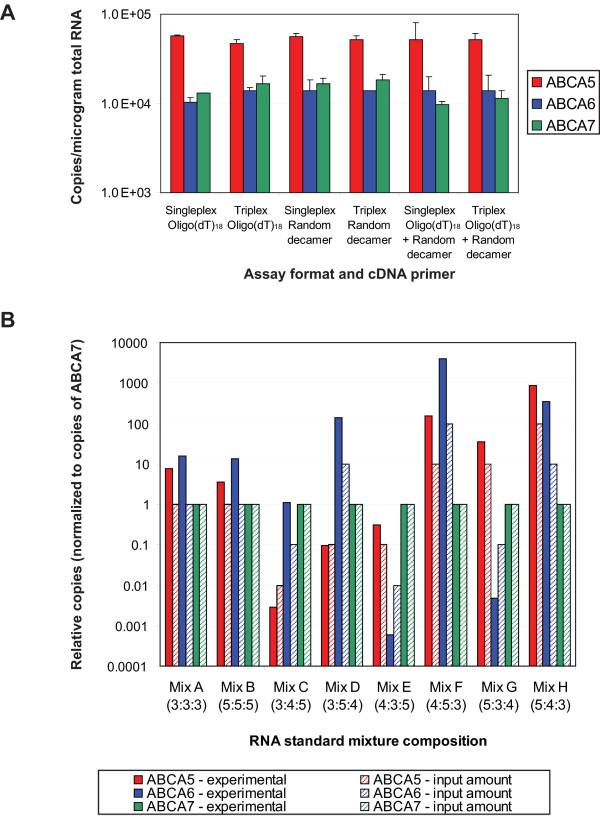
**Real-time one-step RT-PCR evaluation of cDNA priming strategies and the effect of unbalanced target concentrations**. **A) **Real-time one-step RT-PCR evaluation of the effect of different RT primers on quantification of RNA expression in singleplex and in triplex. Reactions employed either an oligo(dT)_18 _RT primer, a random decamer RT primer, or a combination of oligo(dT)_18 _and random decamer RT primers for cDNA synthesis. In addition, each one-step RT-PCR protocol employed *Taq *DNA polymerase, M-MLV RT, 0.8 μg of human thymus total RNA, and CleanAmp™ Precision PCR primers. Reactions were performed in triplicate. **B) **Real-time one-step RT-PCR evaluation of triplex one-step RT-PCR amplifications using different custom prepared mixes containing three RNA standards in different ratios. The relative abundance for each mixture A through H is represented in the following format: (X:Y:Z), where the copies of the ABCA5 RNA standard is present at 10^X copies, the ABCA6 RNA standard is present at 10^Y copies, and the ABCA7 RNA standard is present at 10^Z copies. The observed copy number for each reaction, which was performed in triplicate, was obtained by extrapolation of the Cq to a standard curve for the ABCA5, ABCA6, and ABCA7 RNA standards. The resultant data for each RNA sample was normalized to ABCA7 and was plotted graphically.

To further investigate the factors that may influence the relative quantification of ABCA5, ABCA6, and ABCA7 in a multiplex experiment we first sought to determine whether the cDNA priming strategy would affect quantification. Since the use of oligo(dT)_18 _primers will preferentially amplify the 3' end of transcripts, we next compared reactions which employed oligo(dT) primers, random decamer primers, or a combination of both for the cDNA synthesis step. These one-step RT-PCR studies were performed in triplicate using Precision primers, with singleplex or triplex detection of the three genes in 0.8 μg of human thymus total RNA. When the Cq values were determined, there was very little variation in the calculated values from priming strategy to priming strategy (Figure S3 in Additional file 1). Not surprisingly, the resultant copies of each target determined from extrapolation to a standard curve were similar for singleplex and triplex detection of the ABCA5, ABCA6, and ABCA7 targets (Figure 5A).

Expanding further upon these studies, we next investigated the effect of significantly unbalanced target abundances on one-step RT-PCR performance. Eight mixtures of the ABCA5, ABCA6, and ABCA7 RNA standards were prepared, where each component was present at 10^3^, 10^4^, or 10^5 ^copies/μL. Each of these mixtures was evaluated in triplicate in a triplex real-time one-step RT-PCR experiment, determining the experimental number of copies by extrapolation to a standard curve. For each mixture, the experimentally determined number of copies was typically less than the actual input by an order of magnitude (data not shown). To learn whether the relative abundance of the three targets could be accurately determined in the mix, the determined copies of each of the targets was normalized to that of the ABCA7 target (Figure 5B). While the relative abundances varied from the actual input, in most cases, the trends were maintained. The notable exception in this study was that ABCA6 was detected at higher levels, especially in instances where ABCA6 was one of the more abundant targets. Further analysis of the reaction products by agarose gel electrophoresis revealed that the relative abundances of the three targets in each mix could largely be determined using endpoint analysis (Figure S3 in Additional file 1).

**Figure 6:**
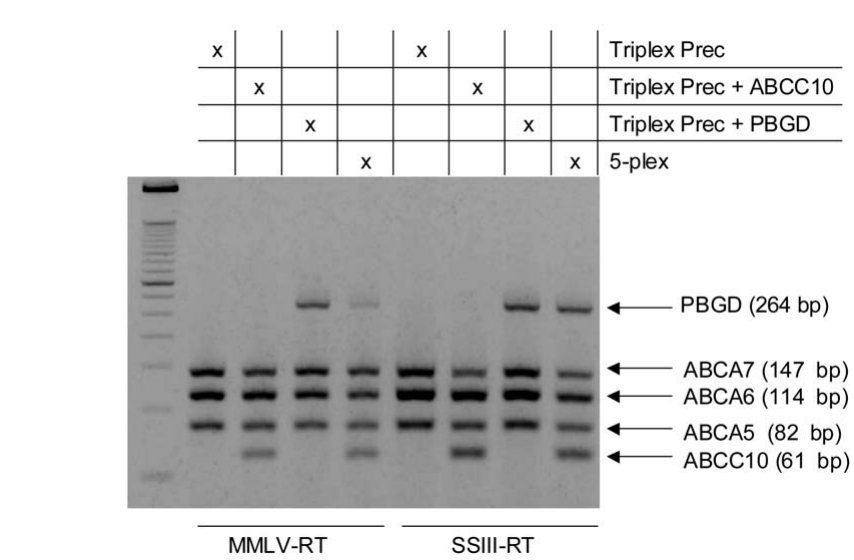
**Evaluation of M-MLV and SSIII reverse transcriptases in multiplex one-step RT-PCR amplification of up to five targets**. The amplification of increasing number of targets was evaluated by using either M-MLV RT (42°C) or SSIII RT (55°C). Reactions contained an oligo(dT)_18 _primer, 0.82 μg of human trachea total RNA, CleanAmp™ Precision primers, and *Taq *DNA polymerase.

In a final set of experiments, conditions were evaluated that would increase the number of targets that could be simultaneously amplified using the modified primers. Using M-MLV as the reverse transcriptase, CleanAmp™ Precision primers supported multiplex amplification of up to five targets (Figure 6), where the unmodified primers could not amplify any of the specific targets. When the more specific reverse transcriptase SSIII RT was used, amplicon formation became more robust, indicating that as the number of targets in a one-step RT-PCR protocol increases, so does the need for a higher temperature cDNA synthesis step to support the downstream PCR amplification.

Overall, the use of primers containing thermolabile protecting group has provided a significant improvement to one-step RT-PCR protocols, allowing for greater specificity in singleplex and multiplex amplification schemes.

## Discussion

Multiplex one-step RT-PCR is a powerful technique that provides several advantages for molecular diagnostics, facilitating genetic screening and analysis of changes in gene expression levels in different tissues among other applications. Performing multiplex RT-PCR in one step instead of two offers a more streamlined process that allows for increased throughput of samples [26]. However, the use of multiple primer pairs in the same reaction requires extensive optimization because amplicons are often amplified with differing efficiencies [27]. Moreover, extension of the PCR primers during the lower temperatures of the reverse transcription step, may result in mispriming and less efficient amplification of the target amplicons. To further improve one-step RT-PCR performance, a number of different approaches have been developed to block the DNA polymerase activity during the RT-PCR step [28] but they require either a significant investment in equipment or a dramatic change in protocols [15,16].

On the other hand, CleanAmp™ Precision primers present a novel approach that relies on the introduction of two thermolabile modification groups into a primer sequence of interest. This approach is much more amenable to existing protocols and does not require additional manipulation steps or the need for additional equipment. These modification groups prevent primer extension during the lower set-up temperatures thereby reducing non-specific amplification. Once the higher temperatures are reached, the thermolabile modifications are released and allow the primers to amplify the desired region of the cDNA product.

The present study describes the benefit of using primers containing thermolabile phosphotriester modification groups (Clean Amp™ Precision primers) in multiplex one-step RT-PCR. Successful simultaneous amplification of three targets was observed in endpoint PCR, in cases where the unmodified primers could not amplify any of the specific amplicons (Figure 2A and Figure 2B). This improved performance using Precision PCR primers is likely the result of the modification blocking the extension of the PCR primers during the RT step of the reaction.

The preparation of RNA standards allowed for evaluation of the quantification of ABCA5, ABCA6, and ABCA7 in real-time PCR, hence permitting a means for relative quantification of expression levels (Figure S2 in Additional file 1). Real-time results reveal that the quantification is not largely affected by the reaction format, as similar copy numbers are determined in both singleplex and multiplex formats. These studies, which encompassed the evaluation of different cDNA priming strategies, different input levels of total RNA, and artificially prepared RNA mixtures with different abundances of the three targets (Figure 4 and 5), gave largely reliable results, with some indication of amplification biases. This bias was most evident for the ABCA6 target, which was observed to vary in some instances, especially when present at high abundance. This phenomenon was consistent with what has been observed in the literature for largely unbalanced expression levels and is therefore not unexpected [14].

Although Precision primers can also be combined in amplification schemes with sophisticated Hot Start DNA polymerases such as Platinum™ *Taq *and AmpliTaq Gold^® ^and with more thermostable reverse transcriptases like SSIII RT, the results are comparable to those obtained with more-affordable non-Hot Start DNA polymerases, M-MLV RT, and CleanAmp™ Precision primers as reaction components (Figure 3). In fact, the amplicon yield decreased when SSIII RT and AmpliTaq Gold^® ^where combined with CleanAmp™ Precision primers, which is most likely due to the synergistic effect of limited release of unmodified primer and unmodified DNA polymerase in the same reaction. Nevertheless, SSIII RT proved to enhance the amplification of more than four targets simultaneously when combined with Precision primers and non-Hot Start *Taq *DNA polymerase (Figure 6). Overall, CleanAmp™ Precision primers provide a significant benefit in multiplex one-step RT-PCR, allowing for cleaner results with the potential for further development as a tool for relative quantification.

## Conclusions

The increasing need for a rapid and accurate way to perform multiplex one-step RT-PCR to diagnose diseases or identify pathogens, makes CleanAmp™ Precision primers a viable choice. The presence of the thermolabile CleanAmp™ primer modifications improves the specificity of cDNA synthesis by blocking the extension of primers during the lower-temperature RT-PCR step. This higher stringency cDNA synthesis allows for more robust amplification of each of the targets, with less interference by primer dimer formation and mispriming. Moreover, the ability to use these modified primers for one-step RT-PCR amplification of multiple targets offers a great opportunity for further development of this technology for use in measuring the relative expression levels of genes in different tissues, both in singleplex and multiplex assays.

## Methods

### Primers and probes

Gene-specific PCR primers for the PBGD, ABCA5, ABCA6, ABCA7, ABCC10 genes were prepared as unmodified and as CleanAmp™ Precision (TriLink BioTechnologies, Inc.), which contains two thermolabile phosphotriester modifications at the 3'-terminal phosphodiester internucleotide linkage and the penultimate phosphodiester internucleotide linkage [19-21]. For each gene, the following primer sequences, which are listed in Table 1, were prepared. Reverse transcription primers used were oligo(dT)_18 _primers (TriLink) or random decamers (Ambion).

**Table 1:** PCR Primer and hydrolysis probe sequences for the five human total RNA targets.

Target (Length)	NCBI Accession Number	Transcript length (nt)	primer and Probe Information			
			
			primer/probe type	Sequence (5'-3')	5' position	Limit of detection
**PBGD ** **(265 bp)**	NM_000190.3	1526	Forward primer	GAGTGATTCGCGTGGGTACC	213	n.d.
				
			Reverse primer	GGCTCCGATGGTGAAGCC	476	
				
			Hydrolysis probe	-	-	

**ABCA5 ** **(82 bp)**	NM_172232.2	8177	Forward primer	GGCTGCTATTCTGACCACTCACTATA	4598	< 7.6E+01 copies
				
			Reverse primer	TTAACTGCCCAGACACCATGAT	4659	
				
			Hydrolysis probe	6 FAM - CAGAGGCTGTCTGTGATCGAGTAGC - BHQ1	4633	

**ABCA6 ** **(114 bp)**	NM_080284.2	5296	Forward primer	CCATGAGAAATGTCCAGTTTCCT	324	<1.6E+01 copies
				
			Reverse primer	TGCTGGGTTAAATTAGATATTGGTGTA	412	
				
			Hydrolysis probe	Alexa Fluor^®^647-TCCTCAGAATCTGGGAAGGGTAGATAAA-BHQ2	355	

**ABCA7 ** **(147 bp)**	NM_019112.3	6832	Forward primer	TTTCTCTGGGACATGTGTAACTACTTG	4981	<8.9E+01 copies
				
			Reverse primer	TGTGATCGACCAGCCATACAG	5104	
				
			Hydrolysis probe	HEX C6-NH- CCTTCCAGCAGAGGGCATATGTG - BHQ1	5042	

**ABCA10 ** **(61 bp)**	NM_033450.2	5118	Forward primer	GCGGGTTAAGCTTGTGACAGA	1601	n.d
				
			Reverse primer	CCCACCCGCAGAACTTGA	1645	
				
			Hydrolysis probe	-	-	

To complement the PCR primer and hydrolysis probe sequences, this table also includes the NCBI Accession Number, the amplicon length, the transcript length, the position along the transcript of the primers/probes, and the lowest limit of detection for each of the RNA standards (ABCA5, ABCA6, and ABCA7).

### One-step RT-PCR (Endpoint)

A one-step RT-PCR protocol was used in which the components were combined in a single tube. Reaction conditions included 1× PCR buffer (20 mM Tris (pH 8.4), 50 mM KCl) (Invitrogen), 1.5 mM MgCl_2 _(Invitrogen), gene-specific PCR primers (0.5 μM) (TriLink), oligo(dT)_18 _primer (1 μM) (TriLink), 0.16 mM dNTPs (New England Biolabs), 0.5 μL of human trachea total RNA or other human total RNA (Stratagene or Ambion, each at ~1.6 μg/μL), 5 U RNase Inhibitor (New England Biolabs), 50 U Moloney Murine Leukemia Virus Reverse Transcriptase (M-MLV RT) (Invitrogen) or SuperScript^® ^III Reverse Transcriptase (SSIII RT) (Invitrogen), and 0.6 U of *Taq *DNA Polymerase, recombinant (Invitrogen) or Platinum^® ^*Taq *DNA Polymerase (Invitrogen) or AmpliTaq Gold^® ^DNA Polymerase (Applied Biosystems), in a 50 μL reaction volume. Thermal cycling conditions utilized a reverse transcription step at 42°C for 30 min when M-MLV RT was used and 55°C for 30 min when SSIII RT was employed; 95°C for 10 min (RT inactivation and initial denaturation step), followed by 45 PCR cycles at 95°C for 30 sec, 60°C for 1 min and final extension at 72°C for 5 min. Experiments were conducted on a Bio-Rad DNA Engine^® ^Thermal Cycler or a Perkin Elmer GeneAmp 2400^® ^thermocycler.

### RNA Standard

RNA standards for ABCA5, ABCA6 and ABCA7 were made by synthesizing the forward gene-specific PCR primer with a T7 promoter sequence (T7-F) and reverse gene-specific PCR primer with a oligo(dT) sequence (dT-R) [29]. Primer sequences for preparation of the RNA standards for ABCA5 (ABCA5-F-T7: 5'-TAATACGACTCACTATAGGGGCTGCTATTCTGACCACTCACTATA and ABCA5-R-dT: 5'-TTTTTTTTTTTTTTTTTTTTTTTTTTTAACTGCCCAGACACCATGAT), ABCA6 (ABCA6-F-T7: 5'-TAATACGACTCACTATAGGCCATGAGAAATGTCCAGTTTCCT and ABCA6-R-dT: 5'-TTTTTTTTTTTTTTTTTTTTTTTTTTGCTGGGTTAAATTAGATATTGGTGTA), and ABCA7 (ABCA7-F-T7: 5'-TAATACGACTCACTATAGGTTTCTCTGGGACATGTGTAACTACTTG and ABCA7-R-dT: 5'-TTTTTTTTTTTTTTTTTTTTTTTTTTGTGATCGACCAGCCATACAG) were synthesized by TriLink BioTechnologies, Inc. PCR products for ABCA5, ABCA6 and ABCA7, which were amplified in one-step RT-PCR experiments using the appropriate primers from Table 1, were diluted 1:100 and were PCR amplified using the respective T7-F and dT-R primers. Reaction conditions were 1× PCR buffer (20 mM Tris (pH 8.4), 50 mM KCl) (Invitrogen) (2.5 mM MgCl_2_) (Invitrogen), T7-F and dT-R primers (0.5 μM), 0.2 mM dNTPs (New England BioLabs), 10 μL of diluted ABCA5, ABCA6 or ABCA7 PCR product, and 5 U *Taq *DNA polymerase (Invitrogen), in a 100 μL reaction volume. Thermal cycling conditions were 95°C for 2 min denaturation step, followed by 30 PCR cycles at 95°C for 30 sec, 60°C for 30 sec, 72°C for 30 sec, and final extension at 72°C for 5 min. PCR amplified products were isolated by QIAquick PCR purification kit (Qiagen) using the manufacturer's recommended protocol. Amplification was verified by gel electrophoresis and the presence of a DNA band of the correct size. RNA standards were made by *in vitro *transcribing ~4 μg of double stranded PCR product by the RiboMAX™ Large Scale RNA Production System-T7 (Promega) using the manufacturer's recommended protocol at 37°C for 4 hrs. The RNA standards were DNase treated using RQ1 RNase-Free DNase (Promega) at 37°C for 15 min to degrade the DNA template. RNA was extracted by the Absolutely RNA^® ^Miniprep Kit (Stratagene) using the manufacturer's recommended protocol for purifying RNA following an enzymatic reaction. The RNA was then gel purified by 6% PAGE and excised from the gel by shaking overnight at room temperature in 500 μL 0.3 M ammonium acetate. After filtration using a 0.2 micron filter to remove the gel slices, ethanol precipitation was performed using 0.1 volume of 3 M sodium acetate and 2.5 volume of cold 100% ethanol. The resultant solution was vortexed briefly and stored at -20°C overnight. Samples were then centrifuged for 30 min, the supernatant was decanted, and the pellets were washed twice with 500 μL of cold 70% ethanol. Pellets were dried at room temperature and resuspended in elution buffer (Stratagene). The RNA standards were analyzed by 6% PAGE to demonstrate that a single RNA band of the correct size was isolated. UV spectrophotometer readings were used to quantitate the absorbance at 260 nm and calculate the number of copies per μL.

### RNA quantification

Total RNA samples were quantified by the fluorescence assay of Quant iT™ RiboGreen RNA reagent (1:2000) (Invitrogen). Samples were excited at 492 nm and the fluorescence emission was measured at 516 nm, with fluorescence readings collected on a Stratagene Mx3005P^® ^QPCR System instrument. Total RNA abundance in μg/μL was determined by extrapolation to a standard curve of the standards provided in the kit. The human total RNA samples from trachea (Stratagene), brain (Ambion), and thymus (Ambion) were quantitated using Quant iT™ RiboGreen RNA reagent to be at 1.64, 1.56, and 1.60 μg/μL, respectively.

### One-step RT-PCR (quantitative real-time)

Quantitative real-time protocols are the same as for endpoint, with the inclusion of hydrolysis probe (0.1 μM) and passive reference ROX dye (30 nM) (Stratagene), in a 25 μL reaction volume. M-MLV reverse transcriptase (25 U) (Invitrogen) and *Taq *DNA polymerase (2.5 U) (Invitrogen) were used in all quantitative real-time experiments. The human total RNA samples evaluated in these studies were from trachea (Stratagene), brain (Ambion), and thymus (Ambion), and 0.5 μL of the stock solution was introduced into the appropriate reactions. The copies of each gene of interest were quantitated by extrapolation to standard curves containing from ~10^1 ^to ~10^8 ^copies of the appropriate RNA standard (prepared as described above) in 100-fold dilutions, where each concentration of RNA standard was performed in triplicate as a singleplex reaction. Serial dilutions of the RNA standards were conducted for each experiment in which copy numbers were determined for a total RNA standard or a synthetic mix of RNA standards at predetermined ratios. Typical amplification plots and resultant standard curves are shown in Figure S2 in Additional file 1. Thermal cycling conditions were 42°C for 30 min (reverse transcription step), 95°C for 10 min RT inactivation and initial denaturation step, followed by 45 PCR cycles at 95°C for 30 sec, 60°C for 1 min. Reactions were performed in a Stratagene Mx3005P^® ^QPCR System instrument.

### Data analysis of one-step RT-PCR (quantitative real-time)

The quantitative real-time RT-PCR protocols described above employed 0.5 μL of commercially-available human total RNA (from brain (Ambion), thymus (Ambion), or trachea (Stratagene)) or 1.0 μL of a synthetic mixture of the three RNA standards for ABCA5, ABCA6, and ABCA7 in predetermined ratios. For generation of standard curves for ABCA5, ABCA6 and ABCA7, serial dilutions of the appropriate RNA standards (~10^8 ^copies, ~10^6 ^copies, ~10^4 ^copies and ~10^2 ^copies) were performed simultaneously in the same experiment. Each RNA standard was amplified in a singleplex one-step RT-PCR which followed the experimental specifics as described in the section entitled "One-step RT-PCR (quantitative real-time)." The standard curve was generated using the MX-Pro software of the Stratagene Mx3005P^® ^QPCR System (Stratagene). The number of copies of each target in the unknown sample was determined by extrapolation to the corresponding standard curve for all experiments except for the experiment represented in Figure 5A, where the standard curve was from a separate experiment. For all experiments where the copies of target were determined, each data point was performed in triplicate.

## Abbreviations

DNA: Deoxyribonucleic Acid; RNA: Ribonucleic Acid; RT-PCR: Reverse transcription PCR; RT: reverse transcription; PCR: polymerase chain reaction; mRNA: messenger RNA; cDNA: complementary DNA; ABC: ATP-binding cassette, SSIII RT: SuperScript^® ^III Reverse Transcriptase; M-MLV: Moloney Murine Leukemia Virus; Taq: Thermus aquaticus; PBGD: Porphobilinogen deaminase; Cq: quantification cycle.

## Competing interests

*Conflict of interest statement*. EHA and NP are employed by TriLink BioTechnologies, Inc. JY was formerly employed by TriLink BioTechnologies, Inc. and contributed to this work while employed here.

## Authors' contributions

NP and JY conceived the study and designed the one-step RT-PCR assay. JY carried out the assay and analyses for detection of up to two targets. EHA optimized amplification of up to five targets and performed the comparative studies with Hot Start DNA polymerases and with more thermostable reverse transcriptases. EHA wrote the first draft of the communication. NP provided additional input for the writing and supervised the study. All authors have read and approved the manuscript.

## Supplementary Material

Additional file 1 **We would also like to include the following additional data file, which is referenced within the publication: 1) Additional File 1 (Word document), which contains four supplementary figures and accompanying text: • Figure S1**. Evaluation of thermolabile primers in one-step reverse-transcription PCR. • Figure S2. Real-time PCR detection of the ABCA5, ABCA6 and ABCA7 RNA standards, using hydrolysis probe detection. • Figure S3. Real-time one-step RT-PCR evaluation of cDNA priming strategies (Cq values). • Figure S4. Comparison of input and observed initial template copy number in real-time triplex one-step RT-PCR. Click here for file
