# Supplementary material for: Selective control of primer usage in multiplex one-step reverse transcription PCR
Source: BMC Mol Biol. 2009 Dec 30;10:113. doi: 10.1186/1471-2199-10-113 (PMC2811113; doi:10.1186/1471-2199-10-113)
Supplement: Additional file 1 — We would also like to include the following additional data file, which is referenced within the publication: 1) Additional File 1 (Word document), which contains four supplementary figures and accompanying text: • Figure S1. Evaluation of thermolabile primers in one-step reverse-transcription PCR. • Figure S2. Real-time PCR detection of the ABCA5, ABCA6 and ABCA7 RNA standards, using hydrolysis probe detection. • Figure S3. Real-time one-step RT-PCR evaluation of cDNA priming strategies (Cq values). • Figure S4. Comparison of input and observed initial template copy number in real-time triplex one-step RT-PCR. [file 1471-2199-10-113-S1.DOC]

## ADDITIONAL FILE 1

## Primers and probes

Gene-specific PCR primers in Table S1 were ordered as unmodified, and CleanAmp™ Precision through TriLink BioTechnologies, Inc. Reverse transcription primers used were either oligo(dT)_18_ primers (TriLink), random decamers (Ambion), or gene-specific primers (TriLink). All reverse transcription primers were prepared as unmodified DNA sequences (Table S2). All hydrolysis probes were prepared with the corresponding fluorophore/quencher (Table S3).

Table S1. PCR Primers and probes

|  | **Forward Sequence 5’ to 3’** | Reverse Sequence 5’ to 3’ | Source |
| --- | --- | --- | --- |
| PBGD  (264 bp) | GAGTGATTCGCGTGGGTACC | GGCTCCGATGGTGAAGCC | TriLink BioTechnologies |
| ABCA1  (205 bp) | GCACTGAGGAAGATGCTGAAA | AGTTCCTGGAAGGTCTTGTTCAC | TriLink BioTechnologies |
| Beta-  actin  (446 bp) | AGAGATGGCCACGGCTGCTT | ATTTGCGGTGGACGATGGAG | TriLink BioTechnologies |
| ABCA5  (82 bp) | GGCTGCTATTCTGACCACTCACTATA | TTAACTGCCCAGACACCATGAT | TriLink BioTechnologies |
| ABCA6 (114 bp) | CCATGAGAAATGTCCAGTTTCCT | TGCTGGGTTAAATTAGATATTGGTGTA | TriLink BioTechnologies |
| ABCA7 (147 bp) | TTTCTCTGGGACATGTGTAACTACTTG | TGTGATCGACCAGCCATACAG | TriLink BioTechnologies |

Table S2. Reverse Transcription Primers (N = A, C, G, T)

|  | Sequence 5’ to 3’ | **Source** |
| --- | --- | --- |
| Oligo(dT)_18_ | TTTTTTTTTTTTTTTTTT | TriLink BioTechnologies |
| random decamers | NNNNNNNNNN | Ambion |
| PBGD reverse | GGCTCCGATGGTGAAGCC | TriLink BioTechnologies |
| ABCA1 reverse | AGTTCCTGGAAGGTCTTGTTCAC | TriLink BioTechnologies |
| Beta-actin reverse | ATTTGCGGTGGACGATGGAG | TriLink BioTechnologies |

**Table S3. Hydrolysis probe sequences**

|  | **Probe Sequence 5’ to 3’** | **Source** |
| --- | --- | --- |
| ABCA5  (82 bp) | 6 FAM - CAGAGGCTGTCTGTGATCGAGTAGC - BHQ1 | TriLink BioTechnologies |
| ABCA6 (114 bp) | Alexa Fluor®647-TCCTCAGAATCTGGGAAGGGTAGATAAA-BHQ2 | TriLink BioTechnologies |
| ABCA7 (147 bp) | HEX C6-NH- CCTTCCAGCAGAGGGCATATGTG - BHQ1 | TriLink BioTechnologies |

**Figure S1. Evaluation of thermolabile primers in one-step reverse-transcription PCR.** For each gene of interest (PBGD, ABCA1 and Beta-actin) [1,2], the PCR primers were unmodified or contained CleanAmp^TM^ Precision modifications. Reactions employed *Taq* DNA polymerase, MMLV RT and 0.5 μg of human brain total RNA. Reverse transcription utilized either an oligo(dT)_18_ primer, a random decamer, or a gene-specific primer.


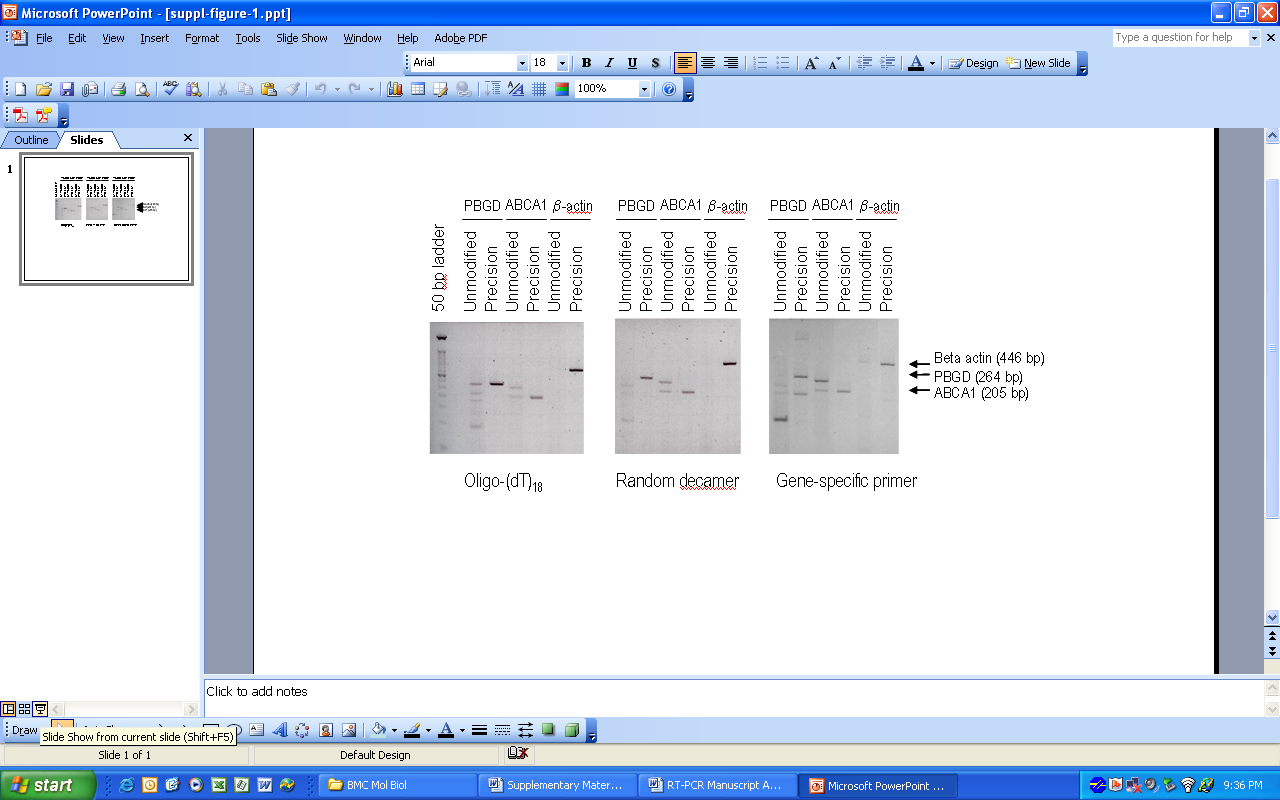


*Experimental Conditions:*

PCR protocols were set up by combining the following components in a single, thin walled 200 μL tube. Components included 1X PCR buffer (20 mM Tris (pH 8.4), 50 mM KCl) (Invitrogen), 1.5 mM MgCl_2_ (Invitrogen), 0.1625 mM dNTPs (New England Biolabs), PCR Primers (0.5 μM) (TriLink), RT Primer (oligo(dT)_18_ primer (1 μM) (TriLink), random decamer primer (1 μM) (Ambion), or reverse PCR primer (1.0 μM) (TriLink)), 1.25 U *Taq* DNA Polymerase (Invitrogen), 0.5 μg human liver total RNA (Ambion) and 50 U of MMLV reverse transcriptase (Invitrogen), in a 50 μL reaction volume. Gene regions of PBGD, ABCA1, and Beta actin, with sizes of 264, 205 and 446 bp respectively, were targeted with PCR primers which were unmodified or contained CleanAmp^TM^ Precision modifications. Reactions that evaluated gene-specific primers for the RT step employed a CleanAmp™ Precision forward PCR primer and an unmodified reverse PCR primer. Thermal cycling conditions were 42°C for 30 min reverse transcription step, 94°C for 10 min followed by 30 PCR cycles at 94°C for 30 sec, 60°C for 30 sec, 72°C for 30 sec and a final extension step of 72°C for 5 min.

*Results:*

The use of CleanAmp^TM^ Precision primers provides better results than unmodified PCR primers for one-step RT-PCR experiments with each of the RT primer types used. Of the three RT primer choices, the use of oligo(dT)_18_ and randomer primers provided the best results. Consequently, Precision primers were the top choice to perform the remainder of the experiments in this manuscript.

**Figure S2.**  **Real-time PCR detection of the ABCA5, ABCA6 and ABCA7 RNA standards, using hydrolysis probe detection.** Reactions were performed in triplicate and contained M-MLV reverse transcriptase, an unmodified oligo(dT)_18_ primer, *Taq* DNA polymerase, CleanAmp™ Precision PCR primers for the ABCA5, ABCA6 and ABCA7 genes, and 10^1^ to 10^8^ copies of the appropriate RNA standard which were prepared as described in the text of the main manuscript. The ABCA5, ABCA6 and ABCA7 amplicons were detected simultaneously using hydrolysis probes labeled at the 5′ end with FAM, CY5, and HEX, respectively. **A)** Amplification plots for the ABCA5, ABCA6 and ABCA7 genes, resulting from reactions that employed ~10^1^ to ~10^8^ copies of the appropriate RNA standard. **B)** Resultant standard curves for the ABCA5, ABCA6 and ABCA7 RNA standards were linear over the entire concentration range. The experimental results for the fit of each standard curve follows (ABCA5, Y = -3.721*LOG(X) + 43.16, Eff. = 85.7%, ABCA6, Y = -3.617*LOG(X) + 43.50, Eff. = 89.0%, ABCA7, Y = -3.942*LOG(X) + 42.08, Eff. = 79.3%).


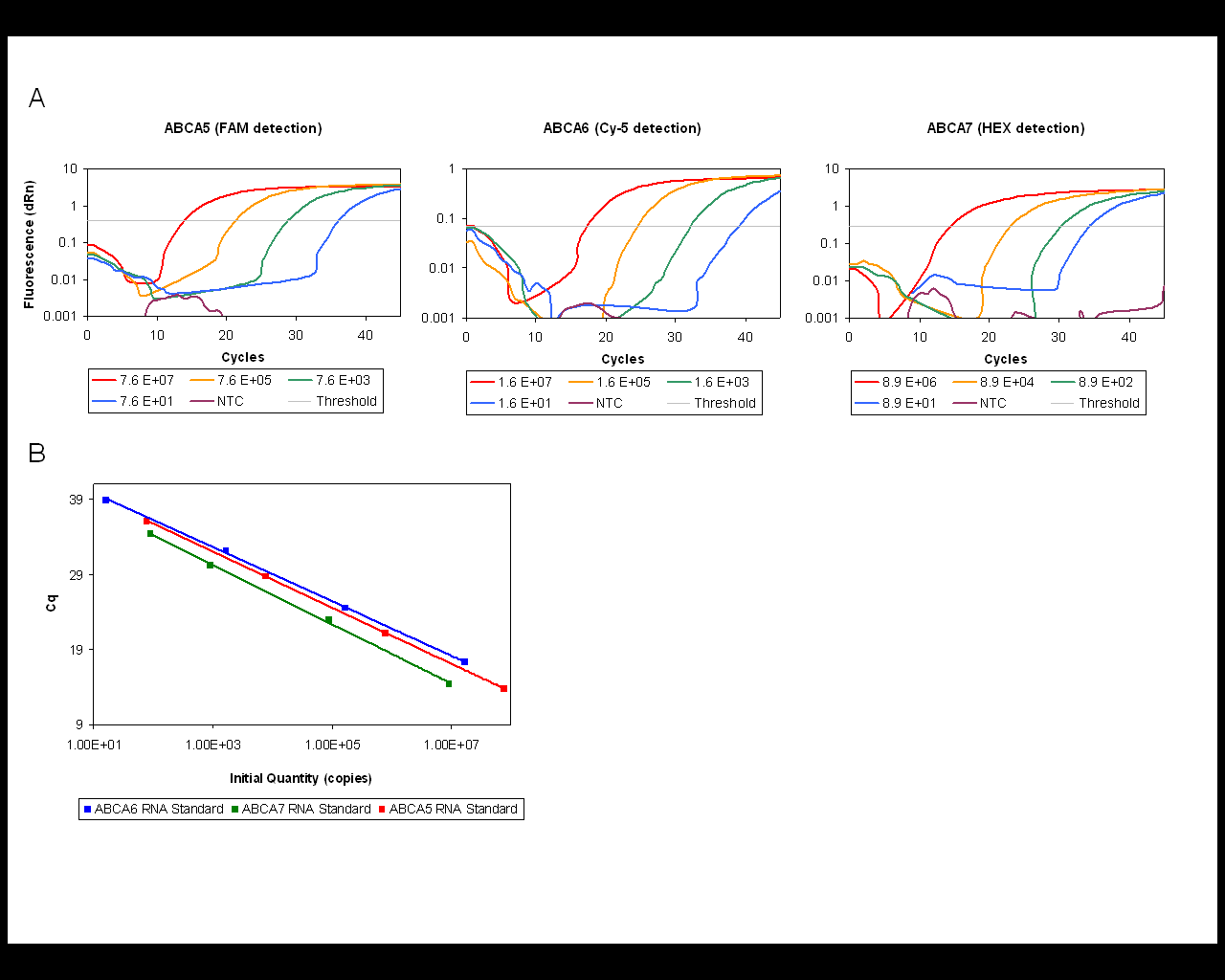


*Experimental Conditions:*

PCR protocols were set up by combining the following components in a single, thin walled 200 μL tube. Components included 1X PCR buffer (20 mM Tris (pH 8.4), 50 mM KCl) (Invitrogen), 1.5 mM MgCl_2_ (Invitrogen), 0.1625 mM dNTPs (New England Biolabs), PCR Primers (0.5 μM) (TriLink), RT Primer (oligo(dT)_18_ primer (1 μM) (TriLink), hydrolysis probe (0.1 μM) and passive reference ROX dye (30 nM), 2.5 U *Taq* DNA Polymerase (Invitrogen), and 25 U of MMLV reverse transcriptase (Invitrogen), in a 25 μL reaction volume. RNA standards for the ABCA5, ABCA6, and ABCA7 targets were amplified at ~ 10^8^, ~10^6^, ~10^4^, and 10^2^ copies/ reaction in singleplex RT-PCR set-ups which included the appropriate PCR primer pair and hydrolysis probe. Amplification of each RNA standard concentration was performed in triplicate. Thermal cycling conditions were 42°C for 30 min reverse transcription step, 95°C for 10 min followed by 45 PCR cycles at 95°C for 15 sec, 60°C for 60 sec on a Stratagene Mx3005P® QPCR System instrument (Stratagene). The resultant amplification plots (Supplementary Figure 2A) and standard curves (Supplementary Figure 2B) were determined using the MX-Pro software provided with the Stratagene Mx3005P® QPCR System instrument.

*Results:*

All three RNA standards are amplified with largely similar efficiencies and were used to quantify the amount of RNA in commercially available total RNA and custom prepared RNA mixtures.

**Figure S3. Real-time one-step RT-PCR evaluation of cDNA priming strategies** (To accompany Figure 5A). Real time one-step RT-PCR evaluation of oligo(dT)_18_ RT primer, random decamer primer and the combination of both RT primers for cDNA synthesis was performed in triplicate. In these studies, a standard curve for the ABCA5, ABCA6, and ABCA7 RNA standards was obtained. Graphical representation of the experimentally determined Cq for 0.8 μg of human thymus total RNA is presented for each sample in singleplex and in triplex. Similar Cq‘s were obtained in singleplex and triplex amplifications regardless of the RT primer used.

*Experimental Conditions:*

Experimental conditions were largely the same as described for Figure S2, with the following exceptions. The RT Primer was either an oligo(dT)_18_ primer (1 μM), a random decamer primer (1 μM), or a combination of an oligo(dT)_18_ and a random decamer primers (0.5 μM each). Human thymus total RNA (0.8 μg) was included as an experimental RNA tissue. The Cq values for amplification of ABCA5, ABCA6, and ABCA7 in human thymus total RNA were determined in singleplex and in triplex. Extrapolation to singleplex standard curves generated using RNA standards allowed for copy number determination.

*Results*

The Cq values are similar for cDNA priming strategy used and for singleplex versus multiplex assay format. Therefore, for these targets RT priming can be performed using oligo(dT)_18_, random decamer and the combination of both RT primers with similar results.

**Figure S4. Comparison of input and observed initial template copy number in real-time triplex one-step RT-PCR.** (To accompany Figure 5B). Custom prepared mixes containing ABCA5, ABCA6 and ABCA7 RNA standards in different concentrations (either 10^3^, 10^4^, or 10^5^ copies of each component represented as 3, 4 and 5 in the figure labels) were amplified to evaluate if the initial relative abundances were conserved after the amplification. Reactions employed oligo(dT)_18_ primer, *Taq* DNA polymerase, MMLV RT, CleanAmp^TM^ Precision primers and 0.5 μl of the RNA Standard mix. Serial dilutions of the three corresponding RNA standards were amplified in singleplex in the same experiment to obtain a standard curve to be used to quantify the abundance of each target.

*Experimental Conditions :*

PCR protocols were set up by combining the following components in a single, thin walled 200 μL tube. Components included 1X PCR buffer (20 mM Tris (pH 8.4), 50 mM KCl) (Invitrogen), 1.5 mM MgCl_2_ (Invitrogen), 0.1625 mM dNTPs (New England Biolabs), Primers (0.5 μM) (TriLink), oligo(dT)_18_ primer (TriLink), Hydrolysis probe (0.1 μM) (TriLink), passive reference ROX dye (30 nM) (Stratagene), 2.5 U *Taq* DNA Polymerase (Invitrogen), 0.5 μl of each RNA standard mix or 0.5 μl of serial dilutions of each RNA Standard, and 25 U of MMLV reverse transcriptase, in a 25 μL reaction volume. Gene regions of ABCA5, ABCA6 and ABCA7 with sizes: 82, 114 and 147bp contained CleanAmp^TM^ Precision modification. Thermal cycling conditions were 42°C for 30 min reverse transcription step, 95°C for 10 min followed by 45 PCR cycles at 95°C for 15 sec, 60°C for 1min.

*Results:*

## Amplification ratios were conserved when the three targets were mixed in equal ratios. When one of the targets in the input mix is in excess, the high abundance target often out-competes the amplification of other targets, especially those targets which are present at low copy number.

## References

1. Kielar, D., W., D., Langmann, T., Aslanidis, C., Probst, M., Naruszewicz, M. and Schmitz, G. (2001) Rapid quantification of human ABCA1 mRNA in various cell types and tissues by real-time reverse transcription-PCR. *Clinical Chemistry*, **47**, 2089-2097.

2. Louwrier, A. and van der Valk, A. (2005) Thermally reversible inactivation of *Taq* polymerase in an organic solvent for application in hot start PCR. *Enzyme and Microbial Technology*, **36**, 947-952.
